# Demonstration of posturographic parameters of squat-stand activity in hemiparetic patients on a new multi-utility balance assessing and training system

**DOI:** 10.1186/1743-0003-10-37

**Published:** 2013-04-15

**Authors:** Rong-Rong Lu, Fang Li, Yi Wu, Yong-Shan Hu, Xiu-Lin Xu, Ren-Lin Zou, Xiu-Fang Hu

**Affiliations:** 1 Department of Rehabilitation, Huashan Hospital, Fudan University, Wulumuqi Middle Road 12, Shanghai, China; 2 School of Medical Instrument and Food Engineering, University of Shanghai for Science and Technology, 516 Jungong Road, Shanghai, China

**Keywords:** Stroke, Squat-stand activity, Posturographic parameters, Visual feedback, Test-retest reliability

## Abstract

**Background:**

Quantitative evaluation of position control ability in stroke patients is needed. Here we report a demonstration of position control ability assessment and test-retest reliability during squat-stand activity on a new system in hemiparetic patients and controls.

**Methods:**

Sixty-two healthy adults and thirty-four hemiparetics were enrolled.

All of the participants were required to complete five repeated squat-stand activities under three different conditions: partial weight support, standard weight bearing, and resistance. The healthy adults’ test was repeated twice to assess the reliability, while the hemiparetics were tested one time to assess impairments in their position control ability. The healthy participants completed their second test 1 wk after the first. Intraclass correlation coefficients (ICCs) were used to assess test-retest reliability.

**Results:**

During partial weight support, the ICCs ranged from 0.77 to 0.91, which indicated a good reliability. During standard weight bearing and resistance, the ICCs varied from 0.64 to 0.86 and 0.54 to 0.84, respectively, indicating a fair reliability. Compared with the healthy adults, the stroke patients demonstrated poorer position control ability.

**Conclusions:**

The posturography of the squat-stand activity is a new and reliable measurement tool for position control. According to the methods proposed here, hemiparetics can be differentiated from healthy adults using the squat-stand activity. This activity will provide a new evaluation tool and therapy with visual feedback for the stroke patients.

**Trial registration:**

Chinese clinical trial registry, ChiCTR-TRC-10000863

## Background

Motor impairment after stroke is a major problem for stroke patients. The risk of falls in hemiparetic patients is dramatically higher than in healthy persons during the rehabilitation period and later during community living [1]. Changing from sitting to standing (sit-to-stand) is an important activity in daily life, and hemiparetics can easily fall during this activity due to a number of impairments. These impairments include low muscle strength, poor position control, and poor sensory input.

Through evaluation, these dysfunctions could be recognized and appropriate training could be carried out. Many evaluations, such as the timed sit-stand-sit test, muscle strength test, static and dynamic balance tests, and other tests, have been used to evaluate the function of hemiparetics. The reliability, validity, and sensitivity of these evaluations have been studied [2-4]. The Berg Balance Scale (BBS) is a classical scale used to evaluate balance. The BBS focuses on whether patients can complete the task and how well they can complete it, but for patients who cannot complete the task, it is difficult to use the BBS for evaluation. It is also important to note that patients cannot complete the task due to a variety of reasons that are not all related to balance impairment. The BBS is similar to the Functional Independence Measure (FIM), Rivermead Motor Assessment, and Rivermead Mobility Index. These tests can only reflect the functional ability of those who can complete the sit-to-stand task [5,6], so it is hard to evaluate the ability of those who cannot, using these evaluation scales. The speed at which a patient completes the timed sit-to-stand test reflects the patient’s ability at this task. In our clinical experience, although some patients can complete the task in a shorter time, many complete the task in an unstable or asymmetrical way, which might lead the patients to suffer a fall easily. Therefore, the timed sit-to-stand test only partially reflects a patient’s position control ability.

For these reasons, we invented a new system to measure position control ability for evaluation in clinical work. Our evaluation system provides back-support via a sliding backboard which enables patients to complete the squat-stand task; the method allows for the patient’s stability and symmetry during the process [7]. These features allow the system to be used in the early stage of the disease to assess those who do not have the ability to complete real sit-to-stand activities. We could also combine the timing of the squat-stand activity and the posturographic parameters to evaluate the ability of patients better. In addition, the static and dynamic balance tests can be used to quantify the balance controlling ability and reaction to the environment [8]. The static balance test assesses stability and symmetry during static standing [9], whereas the dynamic balance test can be used to probe vestibular and brainstem function, including integration of visual input, balance, and position control [10]. These tests cannot be used to quantify position control during the sit-stand-sit process.

In this study, we propose a new system, called the multi-utility balance assessment and training system (MUBATS), that we developed for assessing posturographic parameters during squat-to-stand activity. The MUBATS provides information about abilities such as force production, coordination, and position control. These abilities are necessary in real sit-to-stand activity. Our first goal in assessing the system is to evaluate the reliability of its measurements and its utility in assessing position control in hemiparetic patients.

## Methods

### Participants

A total of 62 healthy adults (23 males and 39 females, mean age 52.48 ± 8.87 y) and 34 stroke patients with hemiplegia (22 males and 12 females, mean age 60.24 ± 12.29 y) were recruited. Healthy participants having any conditions that might affect the assessment protocol, such as cognition, visual, and balance impairments, were excluded from the study. Posturographic parameters of the healthy adults were used to analyze the test-retest reliability. Of the data collected from healthy adults, 34 of the total 64 parameters were selected and used to compare the healthy participants with the hemiparetics in accordance to the age and gender.

For the stroke patients, the following inclusion criteria were applied: motor function of the lower limbs above Brunnstrom level III [11,12]; the ability to complete the sit-to-stand task; and good of tolerance approach. The following exclusion criteria were applied: serious impairments of consciousness, cognition, or emotion that prevented the patient from actively participating in rehabilitation therapy; serious internal disease that requiring limits on exercise; orthostatic hypotension; history of dementia; history of more than a single stroke; advanced osteoarthritis; peripheral neuropathy; unilateral neglect or visual impairment; unwillingness to participate in the research; and inability to participate in follow-up evaluations. All the experimental procedures and the informed consent form signed by the participants were approved by the Ethics Committee of Huashan Hospital (HIRB). This study was conducted in accordance with the Declaration of Helsinki. This trial was registered with chictr.org/cn, number ChiCTR-TRC-10000863.

### MUBATS description

**Figure 1:**
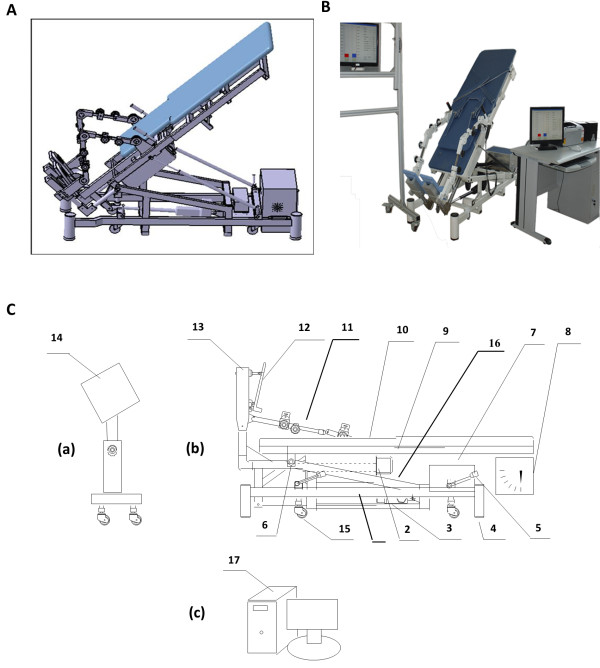
**Design overview of the newly invented MUBATS. A**. Three-dimensional graphic of the MUBATS. **B**. Photograph of the MUBATS. **C**. Sketch designating each component of the MUBATS. The main components are the (**a**) monitor for patients, (**b**) training bed, and (**c**) computer for clinician. The subcomponents are as follows: 1-base; 2 and 3-linear stepper motors; 4-block feet retractable caster; 5-casters handle; 6-connecting shaft; 7-control box; 8-angle display; 9-bed frame; 10-back board; 11-leg support; 12-pedal; 13-pedal bracket; 14-patient monitor; 1 5-casters; 16-bed hoister; and 17-computer.

**Figure 2:**
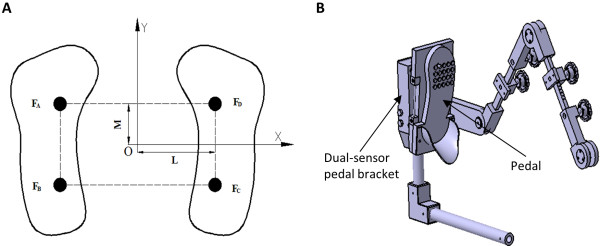
**Force data collection scheme in the MUBATS. A**. Dual platform with separate left and right pedals enabling reacting forces to be evaluated independently on each side. Each pedal has two pressure sensors (black spots). **B**. Plantar pressure testing device.

The newly designed MUBATS was invented by Fudan University and the University of Shanghai for Science and Technology (Figure 1A-C). The system consists of left and right pedals which measure the reacting force (Figure 2A-B), a sensing device, an interface circuit, a data acquisition card, a host computer, a display unit, and a printer. The left and right pedals are separated so that the reacting force on each side can be evaluated separately. Each pedal has two pressure sensors located at the site in contact with the transverse arch and heel, respectively. The testing range of the sensor is 0–100 kg and it has a testing accuracy within 0.5%. In addition, the center of the two pedals coincides with the actual center of the foot pressure according to the zero moment point (ZMP), as described by Vukobratovicli [13]. The software for the system was designed using Visual C++, version 6.0. The process of evaluating and training the patient is shown in the video included with this paper.

### MUBATS procedure

An experienced rehabilitation physician familiar with the MUBATS performed the evaluations. Participants were informed of the aim and given appropriate instructions before the test, in order to help them better understand the study. Posturographic parameters were measured with participants leaning on the oblique slide. Participants had to place their feet on two rectangular pedals in a relaxed and comfortable position. Each participant stood upright, leaned back onto the back board, and then bent his or her knees until the back board would not move any further. After reaching the bottom of the back board’s range, the participant extended his or her knees to return to a standing position. This squat-stand process was repeated five times. The participants were also required to control their center of pressure (COP) by visual feedback of plantar pressure. During the test, participants were instructed to remain as stable and symmetrical as possible.

The test was completed at an angle of 30°, then 45°, and finally 60° (all measured from horizontal as 0°). The participants did the squat-stand activity together with the board, and the board itself weighed approximately 30 kg. As the angle changes, the load carried by the participant versus the machine changes. According to our calculations, at the 30° angle setting, the machine provides partial body weight support (participant bears ~75% of his/her body weight). At the 45° angle setting, there is no net support or resistance (participant bears 100% of his/her body weight). And at the 60° angle setting, the machine transfers net resistance to the participant (participant bears ~125% of his/her body weight). These three angles were tested to see whether the posturographic parameters were reliable under different weight-bearing conditions. The computer software collected planter pressure automatically 20 times per second. The study was performed in a quiet setting with a pleasant visual environment. Before recording commenced, each participant had ~10 s to become familiar with the process. They were instructed to complete the movement cycle several times during this preparation stage. The healthy participants repeated the test twice to assess its reliability, with the second test being conducted one week after the first. The patients were tested only once.

### Posturographic parameters

Plantar pressure was used to evaluate the sit-to-stand activity, and the balance parameters were used to evaluate static balance. In this study, the balance parameters were called posturographic parameters, and they were used to evaluate the squat-to-stand process. The posturographic parameters collected in the test only reflect motor control in the frontal plane because the participants leaned on the oblique and the sensor was located in one dimension in the frontal plane. The parameters include COP, average sway (AS), path length (PL), covered area (CA), maximal sway (MS), and lateral speed (LS).

The parameters were automatically collected and calculated by the computer software. Among them, COP was defined as the mean drift distance projected on the dual platform in the frontal plane. The calculation formula was the same as that published by Genthon [14]. The COP parameter was used to calculate the other parameters. The calculation of the other parameters was the same as for traditional posturographic parameters. Notably, CA was calculated by integrating COP in time in frontal plane, using the following equation:

$$\mathrm{CA}\;=\;\frac{\sum_{i=1}^{n}\pi \left(x_{i}^{2}+y_{i}^{2}\right)}{\mathrm{NTc}}$$

The unit of CA is cm^2^. x_i_ and y_i_ are defined as the coordinates of lateral shift of COP projected on the platform. I = 0, 1, 2……N (I N were integers). Tc is defined as the acquisition time.

### Data analysis

SPSS (version 16.0) was used to perform the statistical analysis in this study. Descriptive statistics were compared using one-way analysis of variance (ANOVA). The Kolmogorov-Smirnov test and F test were chosen to assess the normality and equal variance of the test score. Intraclass correlation coefficients (ICCs) were used to analyze the reliability, which reflected the internal reliability of the evaluation. An ICC < 0.4 indicated poor test-retest reliability. An ICC ranging between 0.4 and 0.75 indicated fairly good test-retest reliability, and an ICC > 0.75 meant there was a good test-retest reliability [15,16].

## Results

Normality and homogeneity tests indicated that the datasets collected had normal distributions. There was no significant difference in variance between the groups (*P* > 0.05). Descriptive statistics of all participants and mean values of all outcomes are presented in Tables 1, 2, 3, 4, 5 and 6.

**Table 1:** Characteristics of participants by group

**Parameter**	**Hemiplegics**	**Healthy adults**
Age (years)	60.24 ± 12.29	52.48 ± 8.87
Gender ratio (male:female)	22:12	20:14
Dominant side (left/right)	0/34	1/33
Damaged brain area (basal ganglia/other)	28/6	/
Affected side (left/right)	18/16	/
Duration of illness (range in days)	30–540	/

**Table 2:** Squat-to-stand test-retest reliability in healthy adults performing the task in the weight-relief condition

**Parameter**	**First time**	**Second time**	**ICC**	**95% ICC confidence interval**
COP (cm)	1.45 ± 1.09	1.47 ± 1.20	0.91	0.82–0.95
AS (cm)	2.29 ± 0.86	2.31 ± 1.04	0.91	0.83–0.95
PL (cm)	36.46 ± 20.28	32.68 ± 18.38	0.77	0.58–0.88
CA (cm^2^)	729.30 ± 405.58	653.52 ± 367.64	0.77	0.58–0.88
MS (cm)	11.62 ± 5.92	9.71 ± 5.17	0.78	0.59–0.88
LS (cm/s)	230.11 ± 114.35	194.18 ± 103.46	0.78	0.60–0.88

**Table 3:** Squat-to-stand test-retest reliability of healthy adults in the standard weight-bearing condition

**Parameter**	**First time**	**Second time**	**ICC**	**95% ICC confidence interval**
COP (cm)	1.37 ± 1.01	1.45 ±1.07	0.80	0.65–0.88
AS (cm)	2.03 ± 0.91	2.17 ± 0.91	0.86	0.76–0.92
PL (cm)	27.23 ± 13.07	26.17 ± 11.28	0.86	0.75–0.92
CA (cm^2^)	544.50 ± 261.41	523.44 ± 225.69	0.86	0.75–0.92
MS (cm)	7.37 ± 3.35	8.23 ± 4.01	0.65	0.39–0.79
LS (cm/s)	147.35 ± 66.97	164.62 ± 80.29	0.64	0.39–0.79

**Table 4:** Squat-to-stand test-retest reliability of healthy adults in the resistance condition

**Parameter**	**First time**	**Second time**	**ICC**	**95% ICC confidence interval**
COP (cm)	0.98 ± 0.72	0.85 ± 0.78	0.83	0.70–0.91
AS (cm)	1.71 ± 0.74	1.58 ± 0.76	0.84	0.71–0.91
PL (cm)	23.51 ± 12.31	18.58 ± 9.61	0.70	0.46–0.83
CA (cm^2^)	470.27 ± 246.13	371.59 ± 192.13	0.70	0.46–0.83
MS (cm)	6.36 ± 3.65	5.74 ± 2.44	0.57	0.24–0.76
LS (cm/s)	125.24 ± 73.29	114.86 ± 48.72	0.54	0.18–0.74

**Table 5:** Posturographic parameters of healthy adults and hemiparetics in the weight relief condition

**Parameter**	**Hemiparetics**	**Healthy adults**	**F**	***P***
COP (cm)	4.23 ± 3.90	1.84 ± 1.20	6.17	0.018
AS (cm)	6.17 ± 3.33	2.76 ± 1.01	17.31	0.000
PL (cm)	2791.10 ± 1429.94	800.89 ± 286.00	33.53	0.000
CA (cm^2^)	137.83 ± 77.74	40.04 ± 14.30	27.55	0.000
MS (cm)	18.47 ± 7.24	11.57 ± 4.98	11.11	0.002
LS (cm/s)	341.67 ± 79.77	230.28 ± 97.55	14.07	0.001

**Table 6:** Posturographic parameters of healthy adults and hemiparetics in the standard weight-bearing condition

**Parameter**	**Hemiparetics**	**Healthy adults**	**F**	***P***
COP (cm)	3.83 ± 3.82	1.51 ± 1.18	11.27	0.001
AS (cm)	5.57 ± 3.57	2.21 ± 1.02	27.45	0.000
PL (cm)	2314.30 ± 1761.87	540.25 ± 267.30	33.65	0.000
CA (cm^2^)	115.71 ± 88.07	27.01 ± 13.37	33.67	0.000
MS (cm)	13.50 ± 4.67	8.20 ± 3.48	26.51	0.000
LS (cm/s)	269.95 ± 93.47	163.90 ± 69.58	26.55	0.000

As reported in Table 1, neither age, gender, nor dominant side differed between the patient and control groups (ANOVA, P > 0.05). Table 2 summaries the posturographic parameters and ICCs in the weight-relief condition (30° angle). The ICCs of COP and AS were over 0.91, indicating very good reliability. The ICCs of the rest of the posturographic parameters were close to 0.77, indicating good reliability.

The posturographic parameters and ICCs in the standard weight-bearing condition (45° angle) are reported in Table 3. The ICCs of COP, AS, PL, and CA were over 0.80, which indicated a good reliability. The ICCs of MS and LS were around 0.65, which indicated a fair reliability. The posturographic parameters and ICCs in the resistance condition are reported in Table 4. The ICCs of COP and AS were over 0.8, indicating good reliability. The ICCs of PL and CA were 0.70, also indicating good reliability. The ICCs of MS and LS were around 0.55, indicating fair reliability. The results reported in Tables 5, 6, and 7 show that the stroke patients had poorer position control ability than did the healthy participants.

**Table 7:** Posturographic parameters of healthy adults and hemiparetics in the resistance condition

**Parameter**	**Hemiparetics**	**Healthy adults**	**F**	***P***
COP (cm)	3.68 ± 3.35	1.31 ± 0.93	8.39	0.007
AS (cm)	4.81 ± 2.78	3.35 ± 5.85	8.39	0.007
PL (cm)	1791.00 ± 1024.35	498.34 ± 253.36	27.01	0.000
CA (cm^2^)	89.55 ± 51.22	24.92 ± 12.67	27.01	0.000
MS (cm)	13.14 ± 4.19	6.45 ± 3.23	28.78	0.000
LS (cm/s)	265.35 ± 84.18	126.49 ± 63.92	31.07	0.000

## Discussion

The central nervous system integrates inputs from the vestibular, somatosensory, and visual systems to maintain stability in space [17]. Most stroke survivors have a combination of sensory, motor, cognitive, and other impairments, leading to dysfunctions when performing basic activities of daily living (ADL). Impaired postural control has the greatest impact on independence in ADL. Desrosiers *et al.*[18] demonstrated that postural control is the best predictor of achieving independent living, and it also shows the highest correlation (r_p_ = 0.70) with person-perceived disability after discharge from a rehabilitation center. Loss of postural control has been recognized as a major problem in stroke patients, which results in a high incidence of falls both during and after rehabilitation, particularly in patients with both motor and sensory deficits. Good position control relates to the effectiveness and safety of activity. In stroke patients, poor stability and poor symmetry of position control not only increase the risk of fall, but also lead to overload of the unaffected side, resulting in a high risk of osteoarticular damage [19,20]. Therefore, improvement of postural control in stroke patients is essential to their independence, social participation, and general health.

The sit-stand-sit test was introduced initially as an outcome measure for assessing lower limb muscle strength [21]. Mong *et al.*[2] demonstrated the reliability and validity of the repeated sit-to-stand test in chronic stroke patients. From a sitting position, horizontal momentum is required to shift the posterior-located center of mass to rise to a standing position. This activity demands relatively good position control ability. Not all patients have the ability to do an unsupported sit-to-stand test, especially in the early stages of recovery. Thus, with the MUBATS, we designed an evaluation system in which stroke patients who do not have the ability to perform the unsupported sit-to-stand test can perform a squat-stand test. The whole evaluation is conducted with the participant leaning against an oblique slide, while simulating the sit-to-stand extension motion. Because the participants can lean on the oblique slide, their squat-stand movement is more stable than in unsupported sit-to-stand activity. Unlike the sit-to-stand movement, the head and body remain aligned during the squat-stand movement. Furthermore, the squat-stand movement in the MUBATS provides back support that confers stability directly. The squat-stand and sit-to-stand movements are similar in that the directions of forces produced and coordination of the hip and knee are nearly the same. Therefore, the squat-stand posturography evaluation should reflect, to some extent, real position control.

Posturography is a method of quantifying the vestibulospinal reflex [22]. In addition to allowing for quantification of postural asymmetry and stability, it also enables us to analyze the recovery course of a patient’s standing posture [23,24]. Posturographic parameters are calculated according to the relationship between the projection of displacement of the center of pressure in the standing plane and time. Meanwhile, COP is calculated as the pressure perceived by ground-pressure pick-up, which equals the reacting force to the ground. A static posturography study, Brière and colleagues found that stroke patients had poor stability in controlling their standing position, and that their COP leans toward the unaffected side [25]. Thus, they proposed the use of dual force platforms to measure the reacting force for the left and right feet.

The reacting force to the ground is the basis for calculating other posturographic parameters. It can also be used as another method to evaluate the sit-to-stand activity [26]. When healthy adults moved their hips away from the seat, the force increased quickly and symmetrically [27]. In contrast, when stroke patients moved from the seat and began to extend, the force increased slowly [28] and the load on the affected side was significantly lower than that of the sound side [29]. A previous study demonstrated the reliability of evaluating stroke patients’ sit-to-stand by vertical ground reaction force [30], and this assessment did not involve repetition times [31]. Until now, few studies attached enough importance to the relationship between the change in load and the time, which is the focus of this study. There may be some potential value in using posturography to evaluate the sit-to-stand movement because it not only quantifies the process of sit-to-stand but also evaluates changes in stability and symmetry before and after rehabilitation.

We tested whether this proposed method was reliable and whether the method could differentiate hemiparetics from healthy patients. The test-retest reliability was one of the statistical methods used to determine the reliability. A high correlation between separate administrations in the test implies good test-retest reliability. Several experiments reported that static posturographic parameters had a good test-retest reliability (ICC > 0.75) [32,33].

The method used in this test is a relatively new one that has not been previously published to evaluate the squat-stand activity [34]. In the weight relief condition, all of the ICCs of the posturographic parameters exceeded 0.75, which indicated good test-retest reliability. Meanwhile, in the standard weight-bearing condition, the ICCs for COP, AS, PL, and CA were over 0.80, but the ICCs for MS and LS were near 0.65. Thus, the former condition showed good test-retest reliability while the latter showed a fair result. In the weight resistance condition, the ICCs for COP and AS were over 0.8, indicating good test-retest reliability. The ICCs for PL, CA, MS, and LS were between 0.54 and 0.70, showing a fair result. From the results, it could be inferred that in the weight-relief and standard weight-bearing conditions, all of the posturographic parameters had good repeatability. In the resistance condition, COP and AS had good repeatability, but PL, CA, MS, and LS had only fair repeatability. COP reflected the center of pressure and body symmetry, whereas AS, PL, and CA moderately reflected position stability. These data could represent position control and had internal reliability. MS and LS absolutely reflected position stability and had no consistency, so the results obtained here could be reasonably explained.

When the posturographic parameters were compared between healthy adults and hemiparetics in three different conditions, the stability and symmetry of hemiparetics were found to be significantly worse than the stability and symmetry of healthy participants. These results demonstrate that this new evaluation method allows the postural control of hemiparetics to be distinguished from that of healthy adults. Accordingly, a therapist could quantitatively measure his or her patient’s position control ability, which may help the therapist know whether a therapy is effective or not.

In this study, we have proposed a new MUBATS method with which to evaluate position control ability during the squat-stand activity. In the first part of the study, we demonstrated the reliability of the method in the healthy population. Our evaluation method is novel compared to previous methods in that it reflects the patient’s position control ability from a different aspect. In our test, the patients repeat a squat-stand five times on an oblique slide. We take the average data from the five repeated squat-stands to evaluate the patient’s ability. Other evaluation methods are based on the premise that the participants should have the ability to complete the sit-to-stand task. If they cannot complete the task, then the results are hard to evaluate. In contrast, our method focuses on how stably and symmetrically the participants can complete the task, and it provides an evaluation and training environment for early stroke patients. In the three conditions we have tested, the system could differentiate the stroke patients from the healthy participants. Therefore, we believe that this new MUBATS method is valid for quantification.

It should be underscored that squat-stand activity is not equivalent to sit-to-stand activity. Only sway in the frontal plane is recorded during squat-stand activity, while motion can occur in multiple planes during sit-to-stand activity. In addition, sit-to-stand is a dynamic, self-perturbing, and complex action that requires proactive strategies and coordination. Hence, sit-to-stand activity requires different motor control pathways than the supported squat-stand movement. Lastly, spasticity and synergies of the limbs may differ substantially between the two tasks; these complications may hinder a more complex unsupported task, but do not interfere with the simpler supported task in the MUBATS.

The present study has some limitations. First, supported squat-stand is not a common real-life activity. These results may not reflect all of the difficulties that can occur in real-life sit-to-stand activity. The squat-to-stand ability may only partly reflect the sit-to-stand ability. Additionally, we use an oblique slide in the evaluation, which makes the activity more stable, and this may not reflect the real sit-to-stand function of hemiparetics. Their real function may be worse than what is measured during the evaluation. Second, the large standard deviations in our data indicate a large variation in personal balance abilities across individuals. Third, the results cannot be generalized to other disease-specific populations because of the subjects’ selection criteria. Finally, while the MUBATS assessment may produce reliable population data, there may be errors in reflecting individuals’ characteristics.

The MUBATS should be tested in more severely compromised subjects and in an earlier phase of rehabilitation. Here, only patients with Brunnstrom level above III were included because we wanted to include only patients that would be able to do the squat-stand task several times so as to ensure reliability of the data as much as possible. Moreover, the patients needed to perform the task across different weight bearing settings (even above their own body weight) to attain the goal of study. In our further research, we will design experiments that expand the testing and training population, particularly to include stroke patients in an early stage of recovery. We will also continue to research the long-term effects of visual feedback training on position control ability.

## Conclusion

Posturography of the squat-stand action is a new method with which to evaluate position control ability in stroke patients. All of the posturographic parameters evaluated in the study can be used to evaluate stable and symmetrical position control. Performance in this test is repeatable in healthy adults. Stroke patients showed more instability and asymmetry than healthy adults. This kind of method has the potential to allow partial evaluation of the sit-to-stand ability in hemiparetic patients.

## Abbreviations

MUBATS: Multi-utility balance assessment and training system; COP: Center of pressure; AS: Average sway; PL: Path length; CA: Covered area; MS: Maximal sway; LS: Lateral speed; ICCs: Intraclass correlation coefficients.

## Competing interests

The authors declare that they have no competing interests.

## Authors’ contributions

RRL carried out the MUBATS evaluations, performed part of the statistical analysis and drafted the manuscript. FL conceived of the study, participated in its design and helped to design the MUBATS. YW participated in the enrollment and evaluation of the patients. YSH performed the statistical analysis and helped to draft the manuscript. XLX designed MUBATS and coordinated in construction of the MUBATS. RLZ programmed the software used in the MUBATS. XFH helped to construct the MUBATS and programmed the software used in MUBATS. All authors read and approved the final manuscript.

## References

[B1] Campbell GB Matthews JT An integrative review of factors associated with falls during post-stroke rehabilitation J Nurs Scholarsh 2010 42 395 404 10.1111/j.1547-5069.2010.01369.x 21091622 PMC4465217

[B2] Mong Y Tilda W Shamay S 5-repetition sit-to stand test in subjects with chronic stroke: reliability and validity Arch Phys Med Rehabil 2010 91 407 413 10.1016/j.apmr.2009.10.030 20298832

[B3] Jin D Yan T Zeng H Validity and reliability of Berg balance scale on assessing balance function Chinese Journal of Rehabilitation Medicine 2003 18 25 27

[B4] Conradsson M Lundin-Olsson L Lindelöf N Littbrand H Malmqvist L Gustafson Y Rosendahl E Berg Balance Scale: Intrarater test-retest reliability among older people dependent in activities of daily living and living in residential care facilities Phys Ther 2007 87 1155 1163 10.2522/ptj.20060343 17636155

[B5] Keith RA Granger CV Hamilton BB Sherwin FS The functional independence measure: a new tool for rehabilitation Adv Clin Rehabil 1987 1 6 18 3503663

[B6] Collen FM Wade DT Robb GF Bradshaw CM The Rivermead mobility index: a further development of the Rivermead motor assessment Int Disabil Stud 1991 3 50 54 1836787 10.3109/03790799109166684

[B7] Berg KO Wood-Dauphinee SL Williams JI Maki B Measuring balance in the elderly: validation of an instrument Can J Public Health 1992 83 suppl 2 7 11 1468055

[B8] Lin D Seol H Nussbaum MA Michael L Madigan. Reliability of COP-based postural sway measures and age-related differences Gait Posture 2008 28 337 342 10.1016/j.gaitpost.2008.01.005 18316191

[B9] Lee WA Deming L Sahgal V Quantitative and clinical measures of static standing balance in hemiparetic and normal subjects Phys Ther 1988 68 970 976 3375321 10.1093/ptj/68.6.970

[B10] Shepard NT The clinical use of dynamic posturography in the elderly Ear Nose Throat J 1989 68 940 943–950,955-957 2620644

[B11] Brunnstrom S Movement Therapy in Hemiplegia: A Neurophysiological Approach 1970 New York: Harper & Row

[B12] Frontera WR Delisa’s Physical medicine and rehabilitation: principles and practice 2010 Fifth Edition Philadelphia: Lippincott Williams & Wilkins 565

[B13] Vulcobratovie M Borvac B Zero-moment point: Thirty five years of its life Int J Humanoid Rob 2004 1 157 173 10.1142/S0219843604000083

[B14] Genthon N Gissot AS Froger J Rouqier P Perennou D Posturography in patients with stroke: estimating the percentage of body weight on each foot from a single force platform Stroke 2008 39 489 491 10.1161/STROKEAHA.107.493478 18174486

[B15] Leroux A Pinet H Nadeau Task-oriented intervention in chronic stroke: changes in clinical and laboratory measures of balance and mobility Am J Phys Med Rehabil 2006 85 820 830 10.1097/01.phm.0000233179.64769.8c 16998429

[B16] Booth ML Owen N Bauman AE Gore CJ Retest reliability of recall measures of leisure-time physical activity in Australian adults Int J Epidemiol 1996 25 153 159 10.1093/ije/25.1.153 8666485

[B17] Ohashi N Nakagawa H Asai M Contribution of vision to the stabilization of body sway in patients with spinocerebellar degeneration Acta Otolaryngol 1993 504 Suppl 117 125 10.3109/00016489309128135 8470515

[B18] Desrosiers J Noreau L Rochette A Bravo G Boutin C Predictors of handicap situations following post-stroke rehabilitation Disabil Rehabil 2002 24 774 785 10.1080/09638280210125814 12437863

[B19] Babyar SR Peterson MG Bohannon R Perennou D Reding M Clinical examination tools for lateropulsion or pusher syndrome following stroke: A systematic review of the literature Clinical Rehabilitatio 2009 23 639 650 10.1177/0269215509104172 19403555

[B20] Martins EF de Araujo Barbosa PH de Menezes LT de Sousa PH Costa AS Is it correct to always consider weight-bearingasymmetrically distributed in individuals with hemiparesis? Physiother Theory Prac 2011 27 566 571 10.3109/09593985.2011.552312 21721993

[B21] Csuka M McCarty DJ Simple method for measurement of lower extremity muscle strength Am J Med 1985 78 77 81 3966492 10.1016/0002-9343(85)90465-6

[B22] Norre ME Forrez G Evaluation of the vestibulospinal reflex by posturography. New perspectives in the otoneurology Acta Otorhinolarynqol Belq 1983 37 679 686 6606931

[B23] Dehail P Petie H Joseph PA Vuadens P Mazaux JM Assessment of postural instability in patients with traumatic brain injury upon enrolment in a vocational adjustment programme J Rehabil Med 2007 39 531 536 10.2340/16501977-0096 17724552

[B24] Oddsson LI Karlsson R Konrad J Ince S Williams SR Zemkova E A rehabilitation tool for functional balance using altered gravity and virtual reality J Neuroeng Rehabil 2007 4 25 31 10.1186/1743-0003-4-25 17623080 PMC1936992

[B25] Brière A Lauzière S Gravel D Nadeau S Perception of weight-bearing distribution during Sit-to-stand tasks in hemiparetic and healthy individuals Stroke 2010 41 1704 1708 10.1161/STROKEAHA.110.589473 20576946

[B26] Mazzà C Stanhope SJ Taviani A Cappozzo A Biomechanical modeling of sit-to-stand to upright posture for mobility assessment of persons with chronic stroke Arch Phys Med Rehabil 2006 87 635 641 10.1016/j.apmr.2005.12.037 16635625

[B27] Carr JH Shepherd RB Stroke rehabilitation—guidelines for exercises and training to optimize motor skill 2002 London: Butterworth-Heinemann 100 120

[B28] Cheng PT Liaw MY Wong MK Tang FT Lee MY Lin PS The sit-to-stand movement in stroke patients and its correlation with falling Arch Phys Med Rehabil 1998 79 1043 1046 10.1016/S0003-9993(98)90168-X 9749681

[B29] Chou SW Wong AM Leong CP Hong WS Tang FT Lin TH Postural control during sit-to-stand and gait in stroke patients Am J Phys Med Rehab 2003 82 42 47 10.1097/00002060-200301000-00007 12510184

[B30] Eng JJ Chu KS Reliability and comparison of weight-bearing ability during standing tasks for individuals with chronic stroke Arch Phys Med Rehabil 2002 83 1138 1144 10.1053/apmr.2002.33644 12161837 PMC3501528

[B31] Hodegs SJ Patrick RJ Reiser RF 2nd Effects of fatigue on bilateral ground reaction force asymmetries during the squat exercise J Strength Cond Res 2011 25 3107 3117 10.1519/JSC.0b013e318212de7b 21993036

[B32] Benvenuti F Mecacci R Gineprari I Bandinelli S Benvenuti E Ferrucci L Baroni A Rabuffetti M Hallett M Dambrosia JM Stanhope SJ Kinematic characteristics of standing disequilibrium: reliability and validity of a posturographic protocol Arch Phys Med Rehabil 1999 80 278 287 10.1016/S0003-9993(99)90138-7 10084435

[B33] Jin D Yan T Tan wen J Reliability of balance performance monitor in the assessment of balance function Chinese Journal of Physical Medicine and Rehabilitation 2002 24 203 205

[B34] Fowler V Carr J Auditory feedback: effects on vertical force production during standing up following stroke Int J Rehabil Res 1996 19 265 269 10.1097/00004356-199609000-00008 8910129

